# Mannose-binding lectin gene polymorphism in psoriasis and vitiligo: an observational study and computational analysis

**DOI:** 10.3389/fmed.2023.1340703

**Published:** 2024-02-09

**Authors:** Mohammed Y. Behairy, Noha Z. Tawfik, Refaat A. Eid, Dalal Nasser Binjawhar, Dalal Sulaiman Alshaya, Eman Fayad, Walid F. Elkhatib, Hoda Y. Abdallah

**Affiliations:** ^1^Department of Microbiology and Immunology, Faculty of Pharmacy, University of Sadat City, Sadat City, Egypt; ^2^Department of Dermatology, Venereology and Andrology, Faculty of Medicine, Suez Canal University, Ismailia, Egypt; ^3^Pathology Department, College of Medicine, King Khalid University, Abha, Saudi Arabia; ^4^Department of Chemistry, College of Science, Princess Nourah bint Abdulrahman University, Riyadh, Saudi Arabia; ^5^Department of Biology, College of Science, Princess Nourah bint Abdulrahman University, Riyadh, Saudi Arabia; ^6^Department of Biotechnology, College of Sciences, Taif University, Taif, Saudi Arabia; ^7^Microbiology and Immunology Department, Faculty of Pharmacy, Ain Shams University, African Union Organization St., Abbassia, Cairo, Egypt; ^8^Department of Microbiology and Immunology, Faculty of Pharmacy, Galala University, Suez, Egypt; ^9^Department of Histology and Cell Biology (Genetics Unit), Faculty of Medicine, Suez Canal University, Ismailia, Egypt; ^10^Center of Excellence in Molecular and Cellular Medicine, Faculty of Medicine, Suez Canal University, Ismailia, Egypt

**Keywords:** psoriasis, vitiligo, MBL, innate immunity, SNP, rs1800450

## Abstract

**Introduction:**

Psoriasis and vitiligo are inflammatory autoimmune skin disorders with remarkable genetic involvement. Mannose-binding lectin (MBL) represents a significant immune molecule with one of its gene variants strongly linked to autoimmune diseases. Therefore, in this study, we investigated the role of the MBL variant, rs1800450, in psoriasis and vitiligo disease susceptibility.

**Methods:**

The study comprised performing *in silico* analysis, performing an observational study regarding psoriasis patients, and performing an observational study regarding vitiligo patients. Various *in silico* tools were used to investigate the impact of the selected mutation on the function, stability, post-translational modifications (PTMs), and secondary structures of the protein. In addition, a total of 489 subjects were enrolled in this study, including their demographic and clinicopathological data. Genotyping analysis was performed using real-time PCR for the single nucleotide polymorphism (SNP) rs1800450 on codon 54 of the MBL gene, utilizing TaqMan genotyping technology. In addition, implications of the studied variant on disease susceptibility and various clinicopathological data were analyzed.

**Results:**

Computational analysis demonstrated the anticipated effects of the mutation on MBL protein. Furthermore, regarding the observational studies, rs1800450 SNP on codon 54 displayed comparable results in our population relative to global frequencies reported via the 1,000 Genomes Project. This SNP showed no significant association with either psoriasis or vitiligo disease risk in all genetic association models. Furthermore, rs1800450 SNP did not significantly correlate with any of the demographic or clinicopathological features of both psoriasis and vitiligo.

**Discussion:**

Our findings highlighted that the rs1800450 SNP on the *MBL2* gene has no role in the disease susceptibility to autoimmune skin diseases, such as psoriasis and vitiligo, among Egyptian patients. In addition, our analysis advocated the notion of the redundancy of MBL and revealed the lack of significant impact on both psoriasis and vitiligo disorders.

## 1 Introduction

Psoriasis and vitiligo are inflammatory autoimmune diseases with genetic factors playing a remarkable role in both diseases (1). Psoriasis is an inflammatory skin disease that is characterized by the development of scaly and erythematous plaques with a global prevalence of approximately 2% (2, 3). Its pathogenesis comprises intricate interactions between the adaptive immune system and the innate immune system (4). Meanwhile, vitiligo represents an autoimmune disease of the skin characterized by autoimmune melanocyte destruction, resulting in the related depigmentation patches affecting the skin in addition to mucosa (5). The prevalence of vitiligo is estimated at approximately 0.2–2% in varied populations (6). Genetic factors have a remarkable role in vitiligo and the risk related to these factors is estimated to reach 75–83%, leaving only approximately 20% for the other environmental factors (7).

One of the important components of innate immunity is mannose-binding lectin (MBL), and the defects of this molecule have been linked with different autoimmune diseases (8). This protein is one of the pattern-recognition molecules that are responsible for activating the complement system through the lectin pathway (9, 10). It is encoded by the *MBL2* gene located on chromosome 10, and the presence of certain missense single nucleotide polymorphisms (SNPs) in this gene was associated with notable low levels of MBL (11). One of these specific mutations is rs1800450 in codon 54, which leads to the change of glycine to aspartic acid (12), with the variant allele represented by allele B and the wild-type one represented by allele A (13). Moreover, this missense mutation could impact the binding activity of MBL, leading to implications on its function (14). The presence of this SNP was linked with several autoimmune diseases such as rheumatoid arthritis (15) and systemic lupus erythematosus (16). However, the relationship between this SNP and autoimmune skin diseases, specifically vitiligo and psoriasis, was investigated by only a few studies showing conflicting results (17–20).

More studies are required to investigate the impact of this missense mutation on psoriasis and vitiligo diseases, especially in light of the increasing interest in the genetic association studies with different human diseases (21–24). By deciphering the nature of the relationship between this mutation and such chronic autoimmune diseases and their features, hopes could be raised to improve the guidelines for the prediction and management of these autoimmune conditions.

## 2 Materials and methods

**Figure 1:**
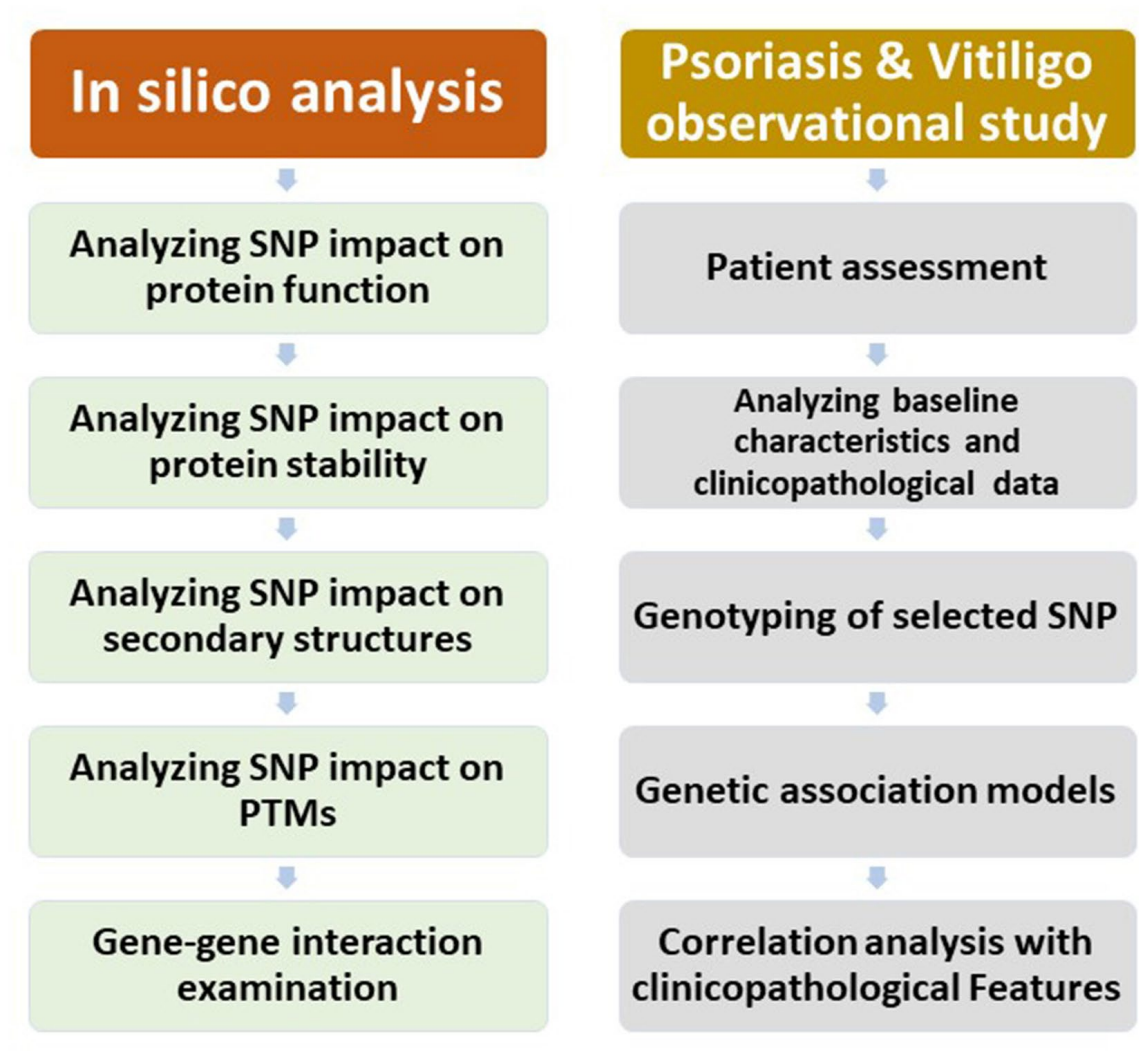
A chart demonstrating the outline of the study work.

The study comprised an *in silico* analysis, an observational study regarding psoriasis patients, and an observational study regarding vitiligo patients. A chart demonstrating the outline of the study research is displayed in Figure 1.

### 2.1 Analyzing the variant’s effect on the MBL protein function

For estimating how the SNP might affect protein function, MutPred2 was used.^1^ MutPred2 represents a software package and machine learning technique that combines genetic and molecular data for reasoning probabilistically regarding the pathogenicity of substitutions of amino acids by providing (1) a general prediction regarding pathogenicity and (2) a list describing certain molecular changes that may affect the phenotype (25).

### 2.2 Analyzing the variant’s effect on the MBL protein stability

The stability of the protein was examined with regard to the chosen mutation using the Mu-Pro tool. The Mu-Pro tool utilizes a strong support vector machine technique that, when used for cross-validation and verification, demonstrates an accuracy of 84%^2^ (26).

### 2.3 Secondary structure analysis

The secondary structure of the studied protein was analyzed using the SOPMA server, and the precise alignment regarding the modified amino acids in the secondary structure was figured out.^3^ In addition, the secondary structures with the selected SNP were examined as well. The SOPMA server could forecast the secondary structures of a given protein by examining the numerous alignments regarding the protein’s sequence (27).

### 2.4 Conducting post-translational modification analysis

The MusiteDeep server^4^ was employed to forecast the positions of various types of post-translational modifications. Due to the significant role of PTMs in controlling how proteins function, the recognition of PTMs is crucial for understanding the pathogenesis of diseases (28–30).

### 2.5 Gene–gene interaction examination

The GeneMANIA tool was employed to produce the *MBL2* gene–gene interaction network.^5^ Using a range of resources and data types, GeneMANIA could predict the genes that strongly interact with a given gene (31).

### 2.6 Protein–protein interaction analysis

The STRING database^6^ was adopted to predict the MBL protein–protein interaction network. The STRING database represents a database into which protein–protein interactions, encompassing both functional and physical relationships, are methodically gathered and integrated (32). The prediction was restricted to the 10 proteins representing the most interactive ones.

### 2.7 Study design of the experimental work

This is a case–control study in which the study participants were recruited from the outpatient dermatology department clinic, Suez Canal University Hospital (SCUH), Ismailia, Egypt, with the study participants classified into three main groups: Group 1 (vitiligo patient group), Group 2 (psoriasis patient group), and Group 3 (control group). All subjects or their next of kin provided informed consent.

#### 2.7.1 Group 1 (vitiligo patients)

After being diagnosed with vitiligo through clinical examination, supplemented with Wood’s lamp, 90 patients from both sexes were included. All the necessary clinicopathological data, such as sex, age, BMI, family history, previous history regarding other autoimmune diseases (e.g., Hashimoto’s thyroiditis, diabetes mellitus, psoriasis, and Addison’s disease), age of the disease onset, severity, disease duration, and treatment, were collected depending on patients’ history. Furthermore, a thorough dermatological examination was applied to all patients to assess the size, site, pattern, and distribution of individual lesions. In addition, disease severity assessment was performed depending on the criteria of the Vitiligo Area Severity Index (VASI) in addition to the Vitiligo European Task Force (VETF).

#### 2.7.2 Group 2 (psoriasis patients)

After being diagnosed with chronic plaque psoriasis, 99 patients of both sexes were included in the study, with the exclusion of those suffering from other autoimmune diseases. All needed clinicopathological data, such as sex, age, BMI, family history, severity, age of the disease onset, lines of treatment, and disease duration, were collected depending on patients’ history. Moreover, a thorough dermatological examination was carried out on all patients to assess the size, site, pattern, and distribution of individual lesions. In addition, disease severity assessment was performed depending on the Psoriasis Area Severity Index (PASI) score. According to the European consensus, PASI is interpreted as mild in cases where the PASI score is less than 10, moderate in cases where the score is between 10 and 20, and severe in cases where the score is greater than 20.

#### 2.7.3 Group 3 (control)

A total of 300 healthy volunteers were recruited from the SCUH blood bank. The members of this third group were matched with the members of the other two groups in terms of sex and age.

### 2.8 Genotyping

The molecular analysis was conducted at the Center of Excellence of Molecular and Cellular Medicine at the Faculty of Medicine, Suez Canal University and Hospital. The extraction of DNA was conducted from venous blood depending on the QIAamp DNA Blood Mini Kit (Cat. No. 51104, QIAGEN; Hilden, Germany). The concentration and purity of DNA were checked depending on the NanoDrop 2000 1^C^ spectrophotometer (NanoDrop Tech., Inc. Wilmington, DE, USA). The DNA samples were stored at −20°C until further analysis. *MBL2* SNP rs1800450 was identified by relying on real-time PCR (RT-PCR) utilizing TaqMan genotyping assays (assay ID: C___2336609_20). The reaction components were obtained from Applied Biosystems (Foster City, CA, USA). A 25-mL reaction volume was used for the PCR, containing 1.25 mL of TaqMan SNP genotyping assay mix, 12.5 mL of TaqMan genotyping master mix, no AmpErase UNG (2×), and 20 ng of genomic DNA, which was diluted to 11.25 mL, utilizing DNase-RNase-free water. After that, the amplification was done using the StepOne™ real-time PCR equipment (Applied Biosystems, Foster City, CA, USA). SDS software version 1.3.1 (Applied Biosystems) was utilized for allelic discrimination. The procedures were conducted blindly with respect to the vitiligo/psoriasis/control groups.

### 2.9 Statistical analyses

For the statistical analysis, we depended on Statistical Package for the Social Sciences (SPSS) software, version 24 in addition to Microsoft® Excel 2010. Percentage and frequency were adopted to express qualitative variables, with the usage of the chi-squared (χ^2^) test besides Fisher’s exact tests to compare between groups. Mean ± standard deviation (SD) was adopted to express quantitative variables, with the use of Student’s *t*-test and one-way ANOVA tests to compare quantitative variables with a normal distribution, while the Mann–Whitney U test and Kruskal–Wallis test were used to compare quantitative variables with a non-normal distribution. Statistical significance was determined by a value of *p* below 0.05. Moreover, the calculation of odds ratios (OR) was performed using a 95% confidence interval (CI). Hardy–Weinberg equilibrium (HWE) was calculated as well. In addition, SNPSTAT was used for genetic models.

## 3 Results

### 3.1 The analysis of the variant’s effect on the MBL protein function

The utilization of the MutPred2 tool revealed a score of 0.849 with rs1800450 SNP, which suggested pathogenicity. Furthermore, molecular alterations were predicted, including losing acetylation at K59 with a probability of 0.27 and a value of *p* of 7.3e-03, gaining SUMOylation at K56 with a probability of 0.25 and a value of *p* of 7.8e-03, losing methylation at K56 with a probability of 0.23 and a value of *p* of 3.1e-03, gaining ubiquitylation at K49 with a probability of 0.17 and a value of *p* of 0.02, and gaining proteolytic cleavage at D53 with a probability of 0.13 and a value of *p* of 0.02.

### 3.2 Analyzing the variant’s effect on the MBL protein stability

The analysis of how the nominated SNP could affect MBL stability was conducted using the Mu-Pro tool. The SNP was predicted by the Mu-Pro tool to decrease the stability with ΔΔG of −0.69653508.

### 3.3 Secondary structure analysis

**Figure 2:**
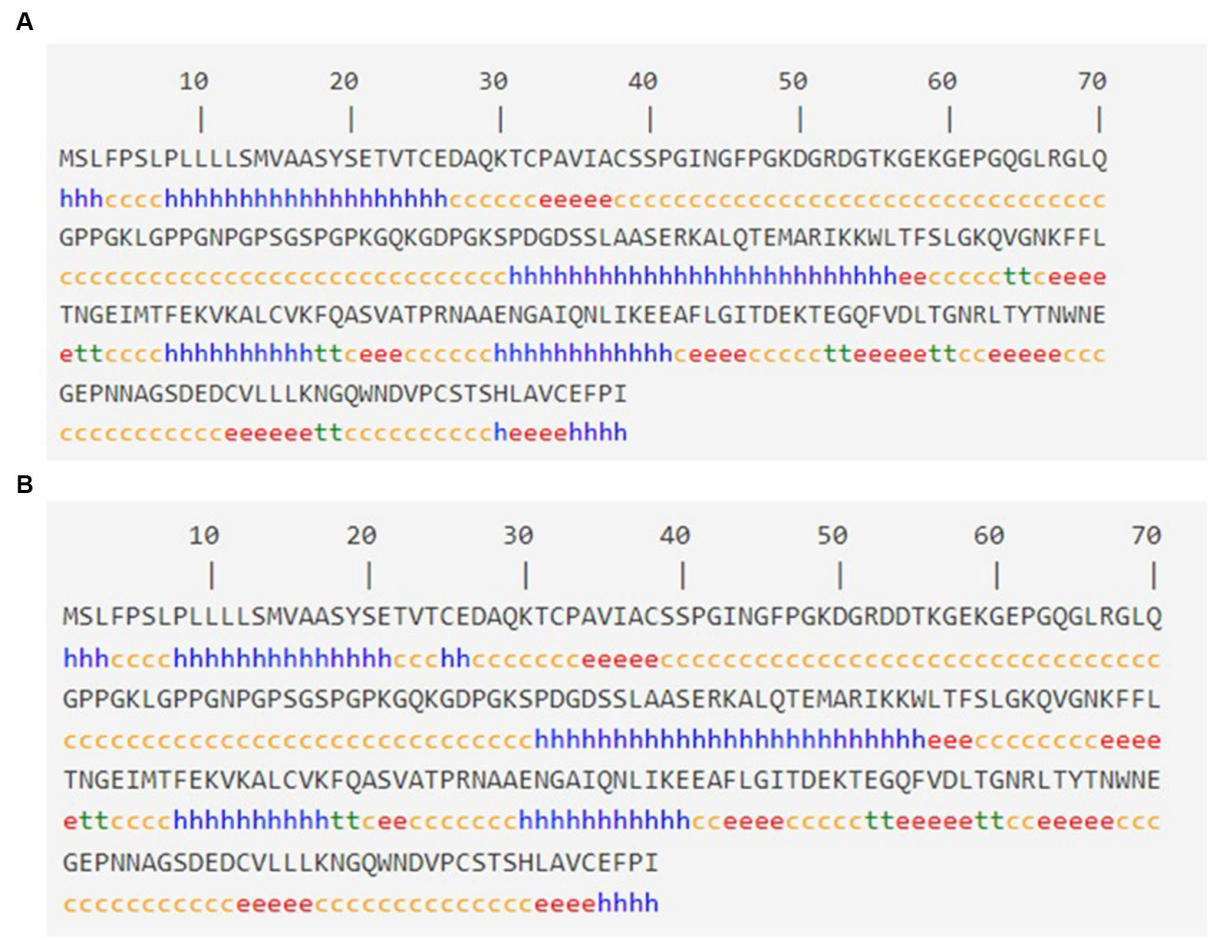
Secondary structure analysis: **(A)** Secondary structure with wild MBL protein and **(B)** secondary structure with MBL protein in case of the selected mutation, produced by the SOPMA server. Alpha helix: (h), Extended strand: (e), Beta turn: (t), Random coil: (c).

The SOPMA tool was used to analyze the anticipated secondary structures of MBL, as demonstrated in Figure 2A. SOPMA revealed that 75 residues were linked with an alpha helix (30.24%), 39 with an extended strand (15.37%), 122 with a random coil (49.19%), and 12 with a beta turn (4.84%). Position 54 was found to be associated with the random coil. Furthermore, the secondary structures with the presence of the selected mutation were predicted, as shown in Figure 2B. In this case, SOPMA exposed that 69 residues were linked with an alpha helix (27.82%), 38 with an extended strand (15.32%), 133 with a random coil (53.63%), and 8 with a beta turn (3.23%). Position 54 was found to be associated with the random coil in this case as well.

### 3.4 The post-translational modification analysis

**Figure 3:**
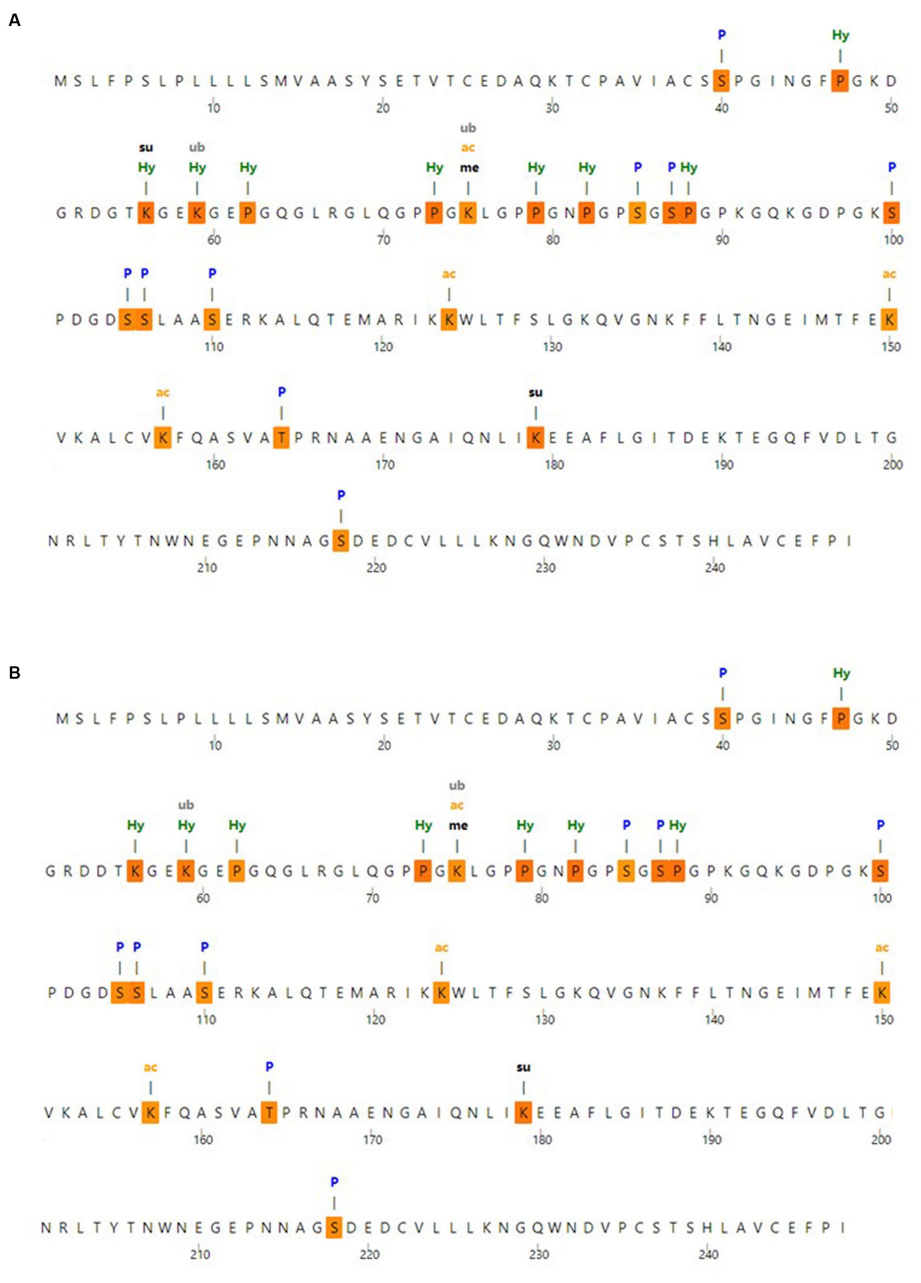
Post-translational modification analysis **(A)** post-translational modification sites with wild MBL protein and **(B)** post-translational modification sites with MBL protein in case of the selected mutation, produced by the MusiteDeep server. P, Phosphorylation; me, Methylation; gl, Glycosylation; Su, SUMOylation; Ub, Ubiquitination; ac, Acetyllysine; pa, Palmitoylation; Pc, Pyrrolidone carboxylic acid; Hy, Hydroxylation.

To identify the expected post-translational modification sites, the MusiteDeep server was utilized; the anticipated post-translational modification sites with MBL protein are shown in Figure 3A. Moreover, the anticipated post-translational modification sites with MBL in the case of the nominated mutation are shown in Figure 3B, and loss of SUMOylation at position 56 could be noticed in this case.

### 3.5 Gene–gene interaction examination

**Figure 4:**
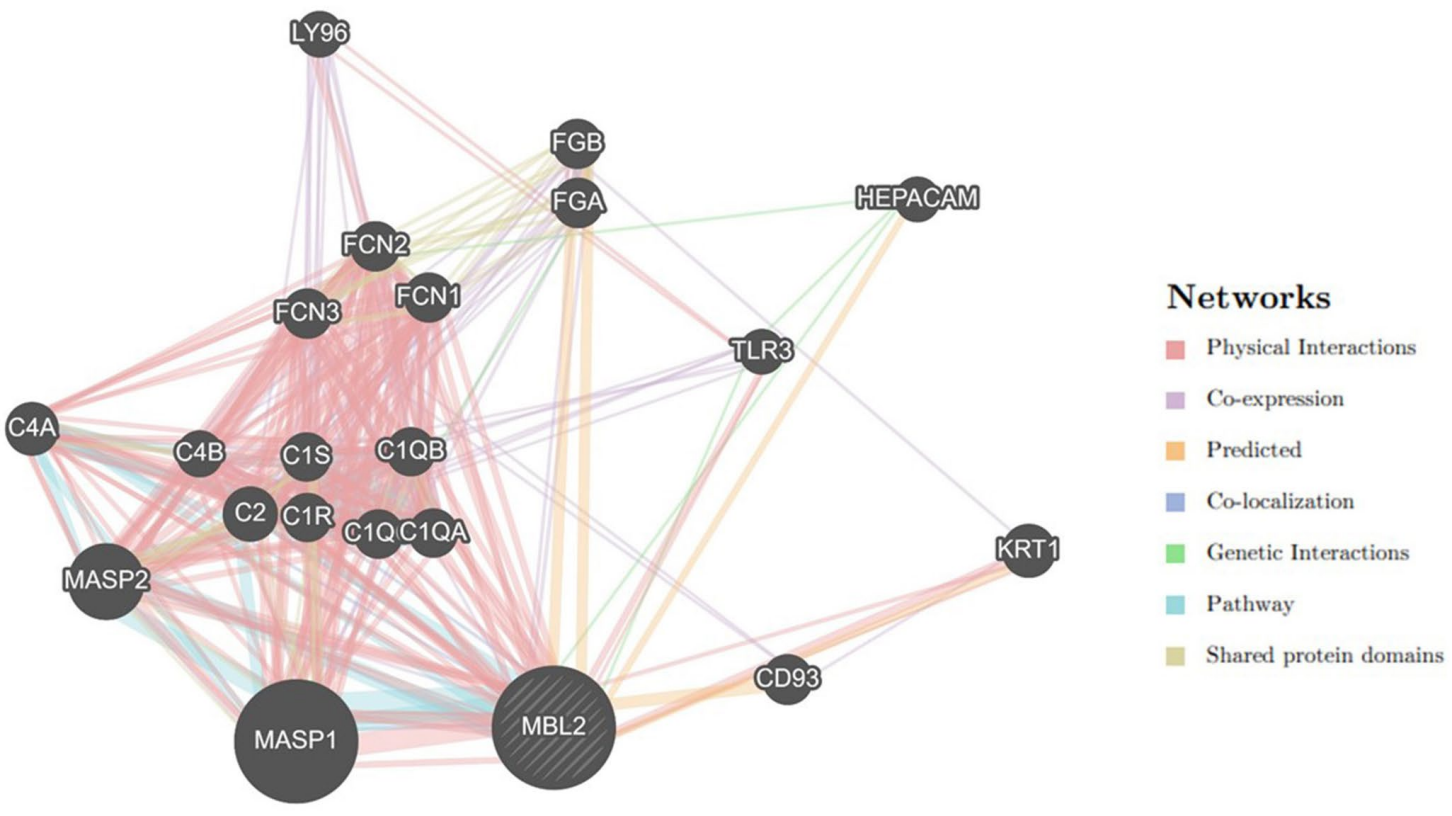
Network of *MBL2* gene–gene interactions, produced by GeneMANIA.

Utilizing the GeneMANIA tool, the *MBL2* gene’s gene–gene interaction was examined, and the results showed 20 genes with the strongest connections to the *MBL2* gene (Figure 4). Among these genes, the mannan-binding lectin serine peptidase 1 gene (MASP1) ranked first. Then, the mannan-binding lectin serine peptidase 2 gene (MASP2) held the second rank. After that, complement C2 (C2) held the third rank.

### 3.6 Protein–protein interaction analysis

**Figure 5:**
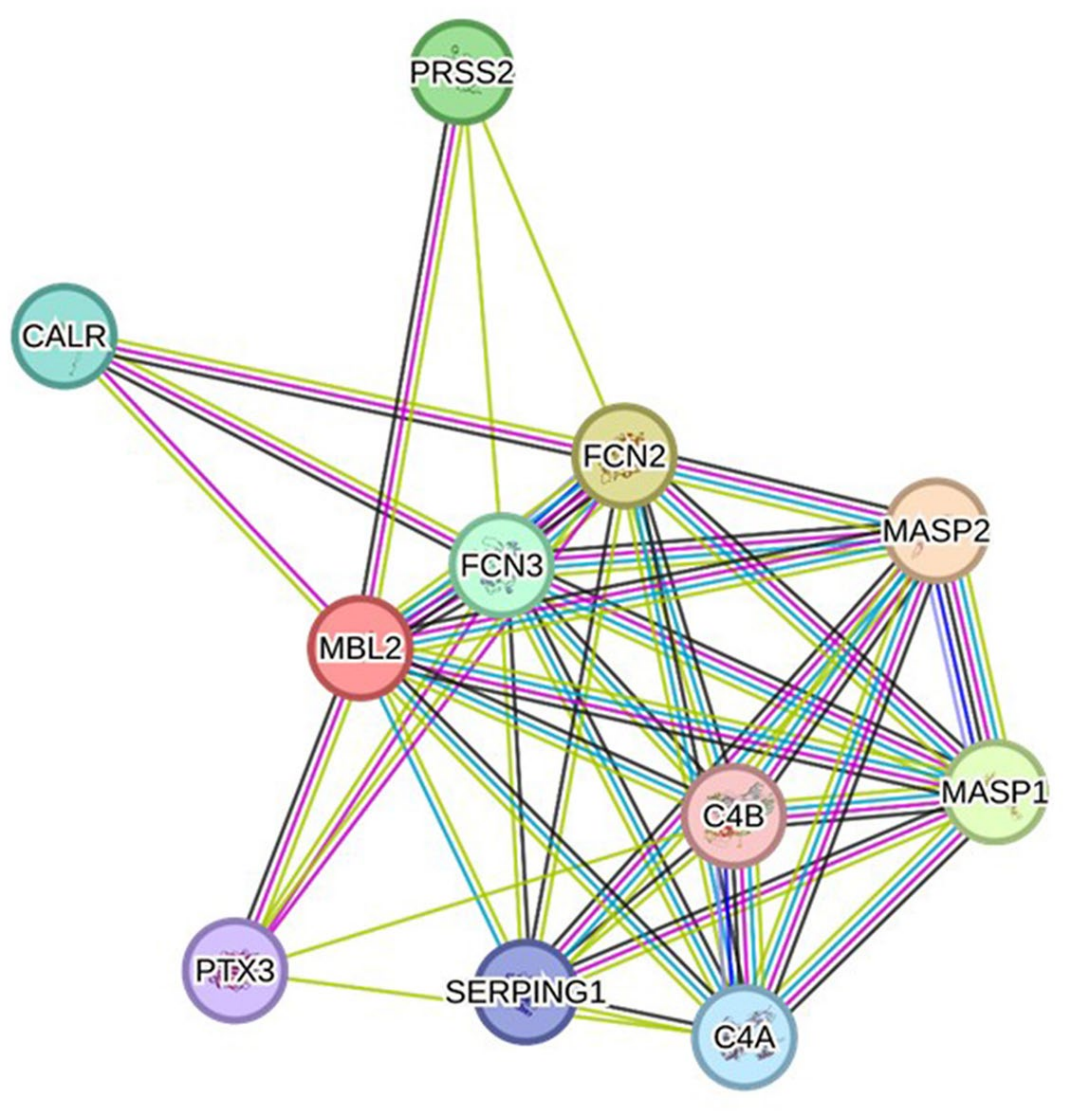
Protein–protein interaction network related to MBL protein, generated by the STRING databases (version 12). The nodes signify proteins, whereas the edges indicate protein–protein associations. The edges could possess any of the seven distinct colored lines, with distinct indications. Black lines: co-expression evidence; light blue lines: database evidence; yellow lines: text-mining evidence; purple lines: experimental evidence; blue lines: co-occurrence evidence; green lines: neighborhood evidence; red lines: fusion evidence. MASP2: Mannan-binding lectin serine protease 2 A chain, FCN2: Ficolin-2, MASP1: Mannan-binding lectin serine peptidase 1, PRSS2: Trypsin-2, FCN3: Ficolin-3, CALR: Calreticulin, C4A: Complement C4-A alpha chain, SERPING1: Plasma protease C1 inhibitor, PTX3: Pentraxin-related protein PTX, C4B: Complement C4-B alpha chain (https://string-db.org/ accessed on 23 December 2023).

Depending on the STRING database, the MBL protein–protein interaction network was generated, and the 10 most interactive proteins were predicted, as could be shown in detail in Figure 5.

### 3.7 Baseline characteristics of population understudy

Our study comprised a total of 489 subjects, including 90 vitiligo patients, 99 psoriasis patients, and a comparable number of healthy control subjects, i.e., 300. The baseline features of enrolled subjects are shown in Table 1. The age of the control, vitiligo, and psoriasis groups ranged from 18 to 63 years, from 6 to 60 years, and from 18 to 60 years, respectively. Meanwhile, the percentage of men in these three groups was 50.3, 61.1, and 45.5%. Considering special habits among the study groups, 53.8% of the control group participants were smokers, 64.4% of vitiligo patients were smokers, and 56.6% of psoriasis patients were smokers. Regarding family history, 18.9% of vitiligo patients had a positive family history, while 15.2% of psoriasis patients had a positive history. No significant statistical difference was found with these baseline characteristics except with the age variable.

**Table 1:** Description of the population understudy.

Variable	Controls (*n* = 300)	Vitiligo (*n* = 90)	Psoriasis (*n* = 99)	Value of *p*
Age, mean (year) *t*	32.0 ± 9.5	34.4 ± 16.6	42.0 ± 14.0	0.001*
Min. – Max.	18–63	6–60	18–60	
Sex M				
Female	149 (49.7%)	35 (38.9%)	54 (54.5%)	0.75
Male	151 (50.3%)	55 (61.1%)	45 (45.5%)	
Smoking M				
Smoker	161 (53.8%)	58 (64.4%)	56 (56.6%)	0.38
Non-smoker	138 (46.2%)	32 (35.6%)	43 (43.4%)	
Family history M				
Positive	**-**	17 (18.9%)	15 (15.2%)	0.562
Negative	**-**	73 (81.1%)	84 (84.8%)	

Data are shown as mean ± SD or number (percentage). A value of *p* of < 0.05 was considered statistically significant. *t*, independent *t*-test between the psoriasis and vitiligo groups (parametric). M, the Mann–Whitney test between the psoriasis and vitiligo groups (non-parametric).

### 3.8 Clinicopathological data among patient groups understudy

Different clinicopathological features between patient groups are shown in Table 2. The mean of disease duration for vitiligo patients and psoriasis patients was 9.2 ± 9.61 and 7.0 ± 6.25, respectively, with no statistical significance. Furthermore, the mean age regarding disease onset for these two groups was 25.93 ± 15.98 and 35.07 ± 13.34, respectively, with statistically significant differences found between these groups. Studying Vitiligo Disease Activity (VIDA) revealed the presence of 37.5, 3.4, 13.6, 20.5, 22.7, and 2.3% of vitiligo patients in stages −1, 0, 1, 2, 3, and 4, respectively, showing statistical significance. Meanwhile, studying psoriasis severity revealed the presence of 45% of psoriasis patients in the mild subgroup, 31% in the moderate subgroup, and 24% in the severe subgroup, with statistical significance found as well. The mean of the Vitiligo Area Severity Index (VASI) was 0.51 ± 0.30 in the vitiligo group, while the mean of the Psoriasis Area Severity Index (PASI) was 13.66 ± 9.05 in the psoriasis group, with statistical significance indicated by a value of *p* less than 0.001.

**Table 2:** Clinicopathological data among patient groups’ understudy.

Variable	Vitiligo patients (*n* = 90)	Psoriasis patients (*n* = 99)	Value of *p*
Disease duration *t*			
Mean ± SD	9.2 ± 9.61	7.0 ± 6.25	0.514
Min. – Max.	0.1–39	1.0–30	
Age of onset *t*			
Mean ± SD	25.93 ± 15.98	35.07 ± 13.34	<0.001*
Min. – Max.	5–59	3–59	
VIDA			
Stage −1	33 (37.5%)		
Stage 0	3 (3.4%)		<0.001*
Stage 1	12 (13.6%)		
Stage 2	18 (20.5%)		
Stage 3	20 (22.7%)		
Stage 4	2 (2.3%)		
Psoriasis severity			
Mild		45 (45%)	0.029*
Moderate		31 (31%)	
Severe		24 (24%)	
VASI *t*			
Mean ± SD	0.51 ± 0.30		<0.001*
Min. – Max.	0.10–1.0		
PASI *t*			
Mean SD		13.66 ± 9.05	<0.001*
Min. – Max.		2.0–41	

Data are shown as mean ± SD or number (percentage). *A significant value of *p* of < 0.05 was considered statistically significant. One-sample chi-squared test was used. *t*, Independent *t*-test between the psoriasis and vitiligo groups (parametric). PASI, Psoriasis Area Severity Index; VASI, Vitiligo Area Severity Index; VIDA, Vitiligo Disease Activity.

### 3.9 *MBL2* genotype analysis

**Figure 6:**
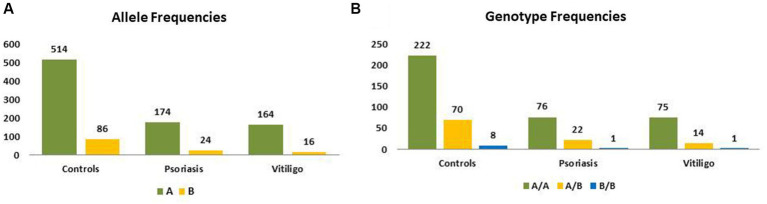
Genotype and allele frequencies of *MBL2* gene rs1800450 SNP among skin autoimmune diseases. **(A)** Allele frequencies and **(B)** genotype frequencies (controls = 300 subjects, psoriasis = 99 patients, vitiligo = 90 patients).

Table 3 shows the genotype and allele frequencies of *MBL2* gene rs1800450 SNP among the studied skin autoimmune diseases. As shown, the allele frequencies in the control group were 514 (85.67%) and 86 (14.33%) for allele A and allele B, respectively. Regarding the psoriasis group, allele frequencies were 174 (87.88%) and 24 (12.12%), respectively, while with the vitiligo group, allele frequencies were 164 (91.11%) and 16 (8.89%), respectively (Figure 6A).

**Table 3:** Genotype and allele frequencies of the *MBL2* gene rs1800450 SNP in the study population.

	*MBL2* (rs1800450)			
	All (*n* = 489)	Control (*n* = 300)	Psoriasis (*n* = 99)	Vitiligo (*n* = 90)
Genotypes				
A/A	373 (76.3%)	222 (74%)	76 (76.8%)	75 (83.3%)
A/B	106 (21.7%)	70 (23.3%)	22 (22.2%)	14 (15.6%)
B/B	10 (2.0%)	8 (2.7%)	1 (1%)	1 (1.1%)
Alleles				
A	852 (87.1%)	514 (85.67%)	174 (87.88%)	164 (91.11%)
B	126 (12.9%)	86 (14.33%)	24 (12.12%)	16 (8.89%)
P HWE	0.74981	0.85667	0.91214	0.93175

Data are shown as numbers (percentage). The Hardy–Weinberg equilibrium was calculated using an online calculator (https://gene-calc.pl/hardy-weinberg-page accessed on 12 July 2023).

**Figure 7:**
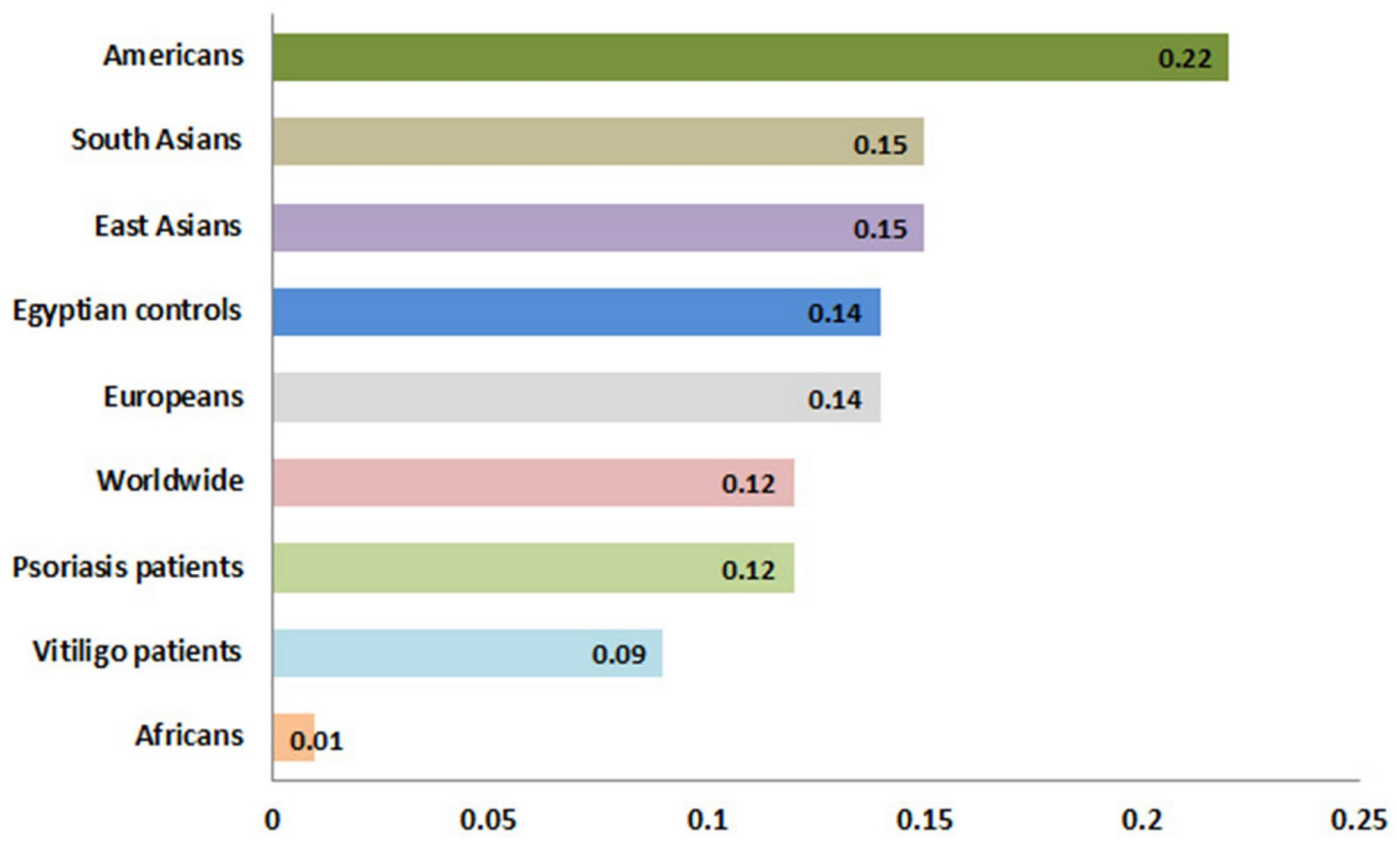
Allele frequencies for *MBL2* gene rs1800450 SNP according to the 1,000 Genomes Project. This diagram was constructed based on Ensembl, https://www.ensembl.org/index.html (accessed on 17 July 2023).

Meanwhile, the genotype frequencies for wild genotype, heterozygous genotype, and mutant genotype with the control group were 222 (74%), 70 (23.3%), and 8 (2.7%), respectively. Regarding the psoriasis group, the genotype frequencies were 76 (76.8%), 22 (22.2%), and 1 (1%), respectively, while with the vitiligo group, the genotype frequencies were 75 (83.3%), 14 (15.6%), and 1 (1.1%), respectively (Figure 6B). The genotype distribution in study groups showed consistency with Hardy–Weinberg equilibrium. In addition, allele frequencies for allele B in different populations for the rs1800450 SNP are shown in Figure 7, according to the 1,000 Genomes project, along with the allele frequencies in the study groups.

### 3.10 Genetic association models analysis

The analysis of *MBL2* gene rs1800450 SNP with psoriasis risk was performed under all genetic association models, as shown in Table 4; nevertheless, no statistical significance was uncovered. In addition, the analysis of the rs1800450 variant with vitiligo risk was conducted under all genetic association models as well; however, no significant statistical difference was found, as shown in Table 5.

**Table 4:** Genetic association models for the *MBL2* gene rs1800450 SNP with psoriasis risk (total *n* = 399, controls = 300, psoriasis patients = 99, adjusted by age and sex).

Model	Genotype	Controls	Psoriasis	OR (95% CI)	Value of *p*
Codominant	A/A	222 (74%)	76 (76.8%)	1	0.7
	A/B	70 (23.3%)	22 (22.2%)	0.99 (0.55–1.79)	
	B/B	8 (2.7%)	1 (1%)	0.42 (0.05–3.80)	
Dominant	A/A	222 (74%)	76 (76.8%)	1	0.83
	A/B – B/B	78 (26%)	23 (23.2%)	0.94 (0.53–1.66)	
Recessive	A/A – A/B	292 (97.3%)	98 (99%)	1	0.4
	B/B	8 (2.7%)	1 (1%)	0.42 (0.05–3.79)	
Overdominant	A/A – B/B	230 (76.7%)	77 (77.8%)	1	0.97
	A/B	70 (23.3%)	22 (22.2%)	1.01 (0.56–1.82)	
Log-additive				0.89 (0.54–1.50)	0.67

CI, confidence interval; OR, Odds ratio; *p*, a value of *p* for comparison between the studied groups.

**Table 5:** Genetic association models for the *MBL2* gene rs1800450 SNP with vitiligo risk (total *n* = 390, controls = 300, vitiligo patients = 90, adjusted by age and sex).

Model	Genotype	Controls	Vitiligo	OR (95% CI)	Value of *p*
Codominant	A/A	222 (74%)	75 (83.3%)	1	0.2
	A/B	70 (23.3%)	14 (15.6%)	0.62 (0.33–1.16)	
	B/B	8 (2.7%)	1 (1.1%)	0.38 (0.05–3.14)	
Dominant	A/A	222 (74%)	75 (83.3%)	1	0.084
	A/B – B/B	78 (26%)	15 (16.7%)	0.59 (0.32–1.09)	
Recessive	A/A – A/B	292 (97.3%)	89 (98.9%)	1	0.37
	B/B	8 (2.7%)	1 (1.1%)	0.42 (0.05–3.44)	
Overdominant	A/A – B/B	230 (76.7%)	76 (84.4%)	1	0.14
	A/B	70 (23.3%)	14 (15.6%)	0.63 (0.33–1.19)	
Log-additive				0.62 (0.35–1.08)	0.074

CI, confidence interval; OR, Odds ratio; *p*, a value of *p* for comparison between the studied groups.

### 3.11 Correlation analysis of the studied variant, psoriasis patients’ demographic data, and clinicopathological features

The inter-relationships between rs1800450 SNP genotyping, psoriasis cases’ demographic data, and their clinicopathological features were analyzed and shown in Table 6. A direct and significant correlation was found between age and duration (*r* = 0.366; *p* < 0.001), disease onset (*r* = 0.890; *p* < 0.001), and disease (*r* = 0.310; *p* < 0.001). Sex had a direct and significant correlation with family history (*r* = 0.890; *p* < 0.05). Moreover, duration had a direct and significant correlation with age (*r* = 0.366; *p* < 0.001), PASI (*r* = 0.381; *p* < 0.001), and severity (*r* = 0.297; *p* = 0.003). Furthermore, PASI showed a direct and significant correlation with duration (*r* = 0.381; *p* < 0.001) as well as with severity and vice versa (*r* = 0.930; *p* < 0.001).

**Table 6:** Correlation matrix between psoriasis patients’ demographic data, clinicopathological features, and the *MBL2* gene rs1800450 SNP genotyping.

		Age	Sex	Smoking	FH	Duration	Disease onset	PASI	Severity	Disease	SNP
Age	*r*	1	−0.024	0.032	−0.247*	0.366**	0.890**	0.157	0.154	0.310**	−0.061
	Value of *p*	-	0.628	0.524	0.014	0	0	0.12	0.128	0	0.22
Sex	*r*	−0.024	1	−0.034	0.216*	0.026	−0.366**	−0.064	−0.036	0.042	0.039
	Value of *p*	0.628	-	0.495	0.032	0.801	0	0.53	0.727	0.401	0.438
Smoking	*r*	0.032	−0.034	1	−0.084	0.013	0.114	0.107	0.121	0.025	−0.025
	Value of *p*	0.524	0.495	-	0.406	0.899	0.262	0.292	0.234	0.617	0.618
FH	*r*	−0.247*	0.216*	−0.084	1	−0.032	−0.230*	−0.011	−0.014	-	0.032
	Value of *p*	0.014	0.032	0.406	-	0.755	0.022	0.911	0.888	-	0.756
Duration	*r*	0.366**	0.026	0.013	−0.032	1	−0.039	0.381**	0.297**	-	−0.041
	Value of *p*	0	0.801	0.899	0.755	-	0.698	0	0.003	-	0.685
Disease onset	*r*	0.890**	−0.366**	0.114	−0.230*	−0.039	1	0.001	0.032	-	−0.079
	Value of *p*	0	0	0.262	0.022	0.698	-	0.99	0.755	-	0.439
PASI	*r*	0.157	−0.064	0.107	−0.011	0.381**	0.001	1	0.930**	-	0.063
	Value of *p*	0.12	0.53	0.292	0.911	0	0.99	.	0	-	0.535
Severity	*r*	0.154	−0.036	0.121	−0.014	0.297**	0.032	0.930**	1	-	0.053
	Value of *p*	0.128	0.727	0.234	0.888	0.003	0.755	0	-	-	0.602
Disease	*r*	0.310**	0.042	0.025	-	-	-	-	-	1	−0.034
	Value of *p*	0	0.401	0.617	-	-	-	-	-	-	0.497
SNP	*r*	−0.061	0.039	−0.025	0.032	−0.041	−0.079	0.063	0.053	−0.034	1
	Value of *p*	0.22	0.438	0.618	0.756	0.685	0.439	0.535	0.602	0.497	-

The correlation coefficient (*r*) represents the value for Spearman’s correlation analysis and its value of *p*s. Shaded boxes enclose values that are statistically significant at either *p* < 0.05 (*) or *p* < 0.01 (**). FH, family history; PASI, Psoriasis Area and Severity Index; SNP, single nucleotide polymorphism.

### 3.12 Correlation analysis of the studied variant, vitiligo patients’ demographic data, and clinicopathological features

The analysis of interrelationships between rs1800450 SNP and vitiligo cases’ demographic data and their clinicopathological features was performed, as shown in Table 7. Age showed a direct and significant correlation with VASI (*r* = 0.271; *p* = 0.01), duration (*r* = 0.438; *p* < 0.001), and disease onset (*r* = 0.812; *p* < 0.001). VASI showed a direct and significant correlation with age (*r* = 0.271; *p* = 0.01) as well as with duration (*r* = 0.447; *p* < 0.001) and vice versa.

**Table 7:** Correlation matrix between vitiligo patients’ demographic data, clinicopathological features, and the *MBL2* gene rs1800450 SNP genotyping.

		Age	Sex	Smoking	FH	VASI	VIDA	Duration	Disease onset	Disease	SNP
Age	*r*	1	0.096	0.039	0.05	0.271**	−0.325**	0.438**	0.812**	0.061	−0.04
	Value of *p*	-	0.058	0.437	0.642	0.01	0.002	0	0	0.233	0.43
Sex	*r*	0.096	1	−0.014	−0.094	0.041	−0.152	0.123	0.135	−0.091	−0.04
	Value of *p*	0.058	-	0.787	0.379	0.699	0.152	0.248	0.205	0.073	0.425
Smoking	*r*	0.039	−0.014	1	0.121	0.021	−0.083	−0.013	0.097	0.092	−0.012
	Value of *p*	0.437	0.787	-	0.255	0.843	0.438	0.907	0.365	0.071	0.818
FH	*r*	0.05	−0.094	0.121	1	0.029	0.058	−0.058	0.039	-	−0.065
	Value of *p*	0.642	0.379	0.255	-	0.788	0.587	0.587	0.713	-	0.542
VASI	*r*	0.271**	0.041	0.021	0.029	1	−0.254*	0.447**	0.068	-	−0.024
	Value of *p*	0.01	0.699	0.843	0.788	-	0.016	0	0.523	-	0.822
VIDA	*r*	−0.325**	−0.152	−0.083	0.058	−0.254*	1	−0.490**	−0.143	-	−0.023
	Value of *p*	0.002	0.152	0.438	0.587	0.016	-	0	0.178	-	0.828
Duration	*r*	0.438**	0.123	−0.013	−0.058	0.447**	−0.490**	1	−0.063	-	0.078
	Value of *p*	0	0.248	0.907	0.587	0	0	-	0.557	-	0.463
Disease onset	*r*	0.812**	0.135	0.097	0.039	0.068	−0.143	−0.063	1	-	−0.045
	Value of *p*	0	0.205	0.365	0.713	0.523	0.178	0.557	.	-	0.675
Disease	*r*	0.061	−0.091	0.092	-	-	-	-	-	1	−0.094
	Value of *p*	0.233	0.073	0.071	-	-	-	-	-	-	0.065
SNP	*r*	−0.04	−0.04	−0.012	−0.065	−0.024	−0.023	0.078	−0.045	−0.094	1
	*p*-value	0.43	0.425	0.818	0.542	0.822	0.828	0.463	0.675	0.065	-

The correlation coefficient (*r*) represents the value for Spearman’s correlation analysis and its value of *p*s. Shaded boxes enclose values that are statistically significant at either *p* < 0.05 (*) or *p* < 0.01 (**). FH, family history; SNP, single nucleotide polymorphism; VASI, Vitiligo Area and Severity Index; VIDA, Vitiligo Disease Activity Score.

## 4 Discussion

Genetics were found to have primary roles in both psoriasis and vitiligo disorders (7, 33–36). SNPs signify the most prevalent type of genetic variation (37), with massive attention attracted to the missense SNP subtype that may result in pathogenic consequences (38). Despite the importance of MBL in the immune system and the discovered links between MBL2 polymorphisms and autoimmune diseases (39), only a few studies to our knowledge have addressed the association between these variants with vitiligo and psoriasis disorders.

By applying the *in silico* methods, the SNP was predicted to affect protein function and protein stability. Furthermore, a protein’s secondary structure plays crucial functions in both the structure and folding of the protein, demonstrating the relevance of this structure (40); therefore, secondary structure analysis was performed using the SOPMA server. The secondary structure analysis detected no change regarding the alignment of the selected position. Moreover, PTMs could influence protein functions and have the potential to impact many aspects of protein biology, such as stability, cellular localization, and interaction with co-factors (41). Therefore, PTM analysis was performed, which revealed a loss of SUMOylation in position 56 with this mutation. In addition, gene–gene interaction examination revealed the 20 genes with the strongest connections to the *MBL2* gene, which might be affected by *MBL2* variants. The significance of researching gene–gene interactions while examining disease–gene associations was demonstrated by the verified presence of interaction between various genetic loci (42). Furthermore, mutations that cause diseases show a tendency to cause instability in protein–protein interactions (43). Consequently, the interaction pattern of MBL in conjunction with other proteins was predicted using the STRING database showing the 10 most prominent interactors.

Our experimental analysis revealed genotype frequencies of 76.3, 21.7, and 2% for AA, AB, and BB, respectively, which were similar to previously conducted studies on Egyptian populations. A 2018 study conducted by Hammad et al. showed genotype frequencies of 79.7, 18.6, and 1.7% with AA, AB, and BB, respectively (44). Moreover, genotype frequencies in another Egyptian study by Gomaa et al. exposed genotype frequencies of 64.6, 28.8, and 6.7%, respectively (12). Although the heterozygous allele displayed a high frequency in these studies along with our study, other previous studies showed even more elevated frequencies with the AB allele (9, 45, 46). With only limited exceptions, these remarkably elevated frequencies of this variant allele and other MBL deficiency variant alleles were detected worldwide. It is noteworthy that the allele B has nearly substituted the allele A in specific Indian populations in South America (47). This distinct pattern has raised inquiries about MBL significance and whether these mutations are associated with some kind of biological benefits (48). Different models were introduced in pursuit of the clarification of this phenomenon, resulting in an unsettled debate (49, 50). One explanation assumed a gained protection with these variants due to the associating low MBL levels that could be advantageous in case of tissue damage resulting from a strong inflammatory response (51). This postulation gave explanation to some previous findings that favored the clinical outcome with genotypes of low MBL in case of some disorders; these genotypes were associated with less severe autoimmune manifestations in primary Sjögren’s syndrome (10); moreover, tuberculosis protection was correlated with heterozygosity in MBL alleles, which is responsible for low levels of MBL (52). Furthermore, similar protective benefit was found for these genotypes against visceral leishmaniasis (53). In addition, the elevated MBL levels were identified to confer an augmented risk of ischemic heart disease along with myocardial infarction in rheumatoid arthritis (54, 55). On the other hand, another explanation excluded the existence of selective pressure and displayed the absence of statistical support for this pressure (56, 57), further advocating the redundancy of MBL in human immunity as a result (57).

Subsequently, the association between codon 54 polymorphism and psoriasis risk was investigated. Nevertheless, no significant association was revealed with all genetic association models. Moreover, no significant correlation was discovered between rs1800450 SNP and the clinicopathological features of psoriasis. The significance of MBL and MBL deficiency in disease pathogenesis has been a source of ambiguity and queries as studies indicate that low or elevated MBL levels show a damaging, protecting, or no effect on susceptibility to diseases (51), which could also be the case here, as recent studies have revealed the important role of MBL in psoriasis pathogenesis. One study found that MBL may aggravate psoriatic skin inflammation by assisting the infiltration of the neutrophils in psoriatic lesions (58). Another study revealed that psoriasis patients displayed higher MBL levels than control subjects and that the level of MBL correlated positively with PASI score and psoriasis severity, and found that MBL assisted the differentiating and infiltrating processes of plasmacytoid dendritic cells in initial skin lesions of psoriasis, leading to the aggravation of disease severity (59). Therefore, the reason for the lack of protective effect with MBL-deficient genotypes needs to be explained. One suggested explanation for the neutrality of MBL deficiency in previous studies was by assuming the redundancy of MBL and the existence of adequate substituting immune mechanisms (9, 60), as other pathways or molecules have demonstrated that they may make up for the deficiency of MBL (57, 61, 62).

The scarcity of previous related studies addressing *MBL2* variants and psoriasis was noticed. The previously conducted study in the Turkish population included 50 psoriasis vulgaris patients along with 53 control subjects and found a significant association between rs1800450 SNP and psoriasis (20), which differs from our findings and with the potential protective role of MBL as well. However, previous analyses related to the impacts of MBL2 variants have pointed out the role of underpowered studies in inconsistent results (63).

In addition, the analysis was extended to examine the relationship between codon 54 polymorphism and vitiligo risk as well. However, the analysis also did not reveal a significant association with all genetic association models. Furthermore, no significant correlation was uncovered between this SNP and clinicopathological features of vitiligo. To our knowledge, only a limited number of previous studies dealt with this topic. Our result complies with the findings of both Karkucak et al. and Dwivedi et al., who investigated this issue; Karkucak et al. conducted their study on a Turkish population comprising 101 vitiligo cases and 101 control subjects, reporting the absence of the association of vitiligo risk with rs1800450 SNP (18). Moreover, Dwivedi et al. conducted their study on an Indian population comprising 92 cases affected by generalized vitiligo and 94 control subjects, revealing the absence of the association of this disease with rs1800450 SNP and other *MBL2* SNPs (17). However, one more Turkish study, including 40 vitiligo patients and 50 healthy subjects, was conducted that suggested a role for rs1800450 in vitiligo susceptibility (19). Complying with the findings of the two studies with a larger sample size gave robustness to our findings. These findings also suggested the notion of redundancy regarding MBL. Finally, the current study had its limitations as well. The patient sample size included in this preliminary study was relatively small. As a result, there is a need for further studies to be conducted on the Egyptian population with a larger patient sample size that encompasses diverse geographic areas. Furthermore, despite investigating the selected variant’s relationship with two autoimmune skin disorders through a combination of experimental and computational approaches, there is still a need for more forthcoming studies that analyze several genetic factors and combine them with environmental factors to understand the full picture regarding these complex diseases.

In conclusion, the *in silico* analysis forecasted the consequences of the presence of the selected mutation on the protein’s function, stability, PTMs, and secondary structures. Moreover, our analysis emphasized the same frequency pattern of the codon 54 variant among the Egyptian population in comparison with other populations and highlighted the unsettled ambiguity regarding this pattern. The studied variant showed no association with the risk of psoriasis and vitiligo as well. Furthermore, this mutation did not significantly correlate with the clinicopathological data for both diseases. These findings supported the redundancy of MBL and the worth of the compensatory mechanisms.

## Data availability statement

The original contributions presented in the study are included in the article/supplementary material, further inquiries can be directed to the corresponding authors.

## Ethics statement

The studies involving humans were approved by the Research Ethics Committee at the Faculty of Medicine, Suez Canal University, Egypt. The studies were conducted in accordance with the local legislation and institutional requirements. Written informed consent for participation in this study was provided by the participants or their next of kin.

## Author contributions

MB: Conceptualization, Investigation, Methodology, Writing – original draft. NT: Investigation, Writing – review & editing. RE: Funding acquisition, Writing – review & editing. DN: Funding acquisition, Writing – review & editing. DA: Funding acquisition, Writing – review & editing. EF: Funding acquisition, Writing – review & editing. WE: Writing – review & editing. HA: Conceptualization, Funding acquisition, Investigation, Methodology, Supervision, Writing – original draft.

## Footnotes

^1^ http://mutpred2.mutdb.org/index.html

^2^ http://mupro.proteomics.ics.uci.edu/

^3^ https://npsa-prabi.ibcp.fr/cgi-bin/npsa_automat.pl?page=/NPSA/npsa_sopma.html

^4^ https://www.musite.net

^5^ http://www.genemania.org

^6^ https://string-db.org/
